# Multi-task learning by using contextualized word representations for syntactic parsing of a morphologically rich language

**DOI:** 10.1371/journal.pone.0332580

**Published:** 2025-09-25

**Authors:** Toqeer Ehsan, Miriam Butt, Sarmad Hussain, Hassan Alhuzali, Ali Al-Laith

**Affiliations:** 1 Quantitative Science and Technology Studies (QSTS), VTT Technical Research Centre of Finland, Espoo, Finland; 2 Department of Linguistics, University of Konstanz, Konstanz, Germany; 3 Center for Language Engineering (CLE), Al-Khawarizmi Institute of Computer Science (KICS), University of Engineering and Technology (UET), Lahore, Pakistan; 4 Department of Computer Science and Artificial Intelligence, Umm Al-Qura University, Makkah, Saudi Arabia; 5 Computer Science Department, Copenhagen University, Copenhagen, Denmark; University of Sargodha, PAKISTAN

## Abstract

We address the challenge of syntactic parsing for Urdu, a morphologically rich language, and present state-of-the-art results for both constituency and dependency parsing. This paper offers four major contributions: 1) the conversion of the CLE-UTB phrase structure treebank into a dependency treebank by developing language-specific head-word and phrase-to-dependency label mapping rules; 2) a novel sequence labeling scheme that transforms the parsing task into a unified representation; 3) the training of contextualized word representations on a large 220 million tokens Urdu corpus collected from the web; and 4) development of parsing framework using two learning paradigms, single-task and multi-task learning. Several post-processing rules are applied to improve the quality of the automatically converted dependency structure treebank. The proposed sequence labeling scheme enables the use of a shared architecture that learns the syntactic structures from both grammatical structures simultaneously and hence improves generalization. Experiments show that the multi-task learning setup significantly enhances parsing performance, achieving an F1 score of 91.39 for constituency parsing (an improvement of 3.29 points) and a labeled attachment score of 85.69 for dependency parsing (an improvement of 1.49 points). These results demonstrate that learning cross-task representations provides measurable benefits and advances the state of syntactic parsing for Urdu.

## 1 Introduction

Urdu is a morphologically rich language, written in a version of the Arabic script from right to left. As an Indo-Aryan language, it is mainly spoken in the South Asian region. There are over 160 million speakers worldwide who use Urdu as their first and second language [1]. It holds several significant linguistic properties that include rich morphology [2], a complex case system [3,4], complex predicate structure [5,6] and flexible word-order [7]. The distinctive characteristics of Urdu pose challenges for natural language processing. Despite being considered a low resource language, a number of efforts have been undertaken in the past years to develop essential linguistic resources, enabling the performance of various NLP tasks. These include the creation of Urdu corpora, morphological analysis [2,8], tokenization and word segmentation [9,10], part of speech (POS) tagging [11,12], sequence chunking [13] and syntactic parsing [14–17]. Urdu remains under-resourced despite these efforts emphasizing the need for continued development of linguistic resources and tools to achieve computational results that can compete with more linguistically resourced languages.

A phrase=structure (PS) treebank illustrates the constituency structure of clauses and their hierarchical organization in a sentence. The information of arguments is encoded at phrase level in the form of linear order. A PS parser provides the information of clauses in sentences, but it lacks grammatical relations. However, functional labels can be attached to phrase labels to represent grammatical roles. On the other hand, the dependency=structure (DS) focuses on functional dependencies between lexical items in clauses and dependency relations between constituents. It highlights grammatical relations between predicates and their arguments which are essential to extract the information about event participants in the applications of natural language understanding (NLU). Urdu has both types of treebanks available that vary in size and annotation schemes. A promising approach is to convert existing PS treebanks into common DS. This conversion aims to facilitate the training of high-quality parsers that can capture both constituency and dependency information. Treebanks are essential resources for a range of NLP tasks. Several treebanks have been developed using the PS [18–22] and the DS [23–26]. The development of the Penn Treebank gave new perspectives and inspirations for the creation of syntactic resources for other languages and corpus-based analysis.

Multi-task learning is an approach that involves training a single model to handle multiple related tasks simultaneously. It leverages common knowledge and cross-representations to improve overall performance. In recent years, multi-task learning has found applications in various natural language processing tasks [27] and demonstrated its applicability in sentiment analysis [28,29], named entity recognition [30,31], semantic role labeling [32], extraction of biomedical relations [33], polarity classification [34], sarcasm and toxic comments detection [28,35], speech recognition [36] and syntactic parsing [37,38]. In this work, we present the conversion of an Urdu PS treebank [16] into a DS, utilizing the Universal Dependencies (UD V2) label sets. To achieve an equivalent DS, we devised a head-word model and created a mapping from phrase labels to dependency labels. In addition, various post-conversion rules have been employed to ensure accurate conversion. In natural language parsing, constituency and dependency parsing are considered disjoint tasks and executed independently [39–42]. However, there is potential to improve both constituency and dependency parsing by incorporating cross-structure representations into the learning process [37,38].

For the constituency structure, we enhanced CLE-UTB labeling scheme, resulting in improved scores. Concerning the DS, we employed the sequence labeling approach from [43], initially introduced in [44]. Prior to multi-task learning, we performed single-task learning-based parsing experiment to achieve the results for constituency and dependency parsing independently. We developed a neural parsing framework that is based on Long Short-Term Memory (LSTM) networks. Bidirectional LSTM (BLSTM) networks have proven to be highly effective in solving sequence labeling problems. The multi-task learning paradigm transforms the constituency and dependency parsing into a sequence labeling problem aligning well with the capabilities of BLSTM networks for neural modeling. Various features have been incorporated into the experiments including character embeddings and convolutions, POS tags, and pre-trained word representations. To perform transfer learning, we trained two types of word representations, context-free embeddings [45] and deep contextualized word representations (ELMo) [46]. The single-task constituency parser achieved the highest F-score of 90.94 when trained with three hidden LSTM layers along with POS tags and embeddings from the third layer of ELMo representations. Similarly, the single-task dependency parser obtained a labeled attachment score (LAS) of 85.36 incorporating POS tags, Word2Vec and ELMo embeddings. The multitask learning-based parser achieved an F-score of 91.39 with an improvement of 3.29 points compared to the state-of-the-art score for the CLE-UTB and with an improvement of 3.53 points for function labeling accuracy. The multi-task dependency parser improved the parsing scores by 1.49 points and achieved a labeled attachment score of 85.69. The single-task constituency and dependency scores presented in this work are state-of-the-art results for the CLE-UTB which are further improved by learning cross-structure representations by employing multi-task learning.

The remainder of the paper is organized as follows. Sect 2 briefly describes the compatibility of the PS CLE-UTB with the DS and related work. Sect 3 presents the conversion approach by providing the details of head-word model, phrase-to-dependency label mappings, and post-conversion rules. Sect 4 describes the sequence labeling schemes for both PS and DS treebanks. Sect 5 presents neural parsing models that include single-task and multi-task learning models along with the transfer learning approach. Sect 6 presents parsing results and their interpretation, and the conclusion of the work is presented in Sect 7.

## 2 Related work

Currently, there are two prominent PS treebanks available for Urdu, Urdu.Kon-TB [47] and CLE-UTB [16]. Both treebanks are quite different with respect to their structure, annotation scheme, annotation depth, genre coverage, and size. This section provides analysis of their structure and suitability for multi-task learning parsing.

**Fig 1:**
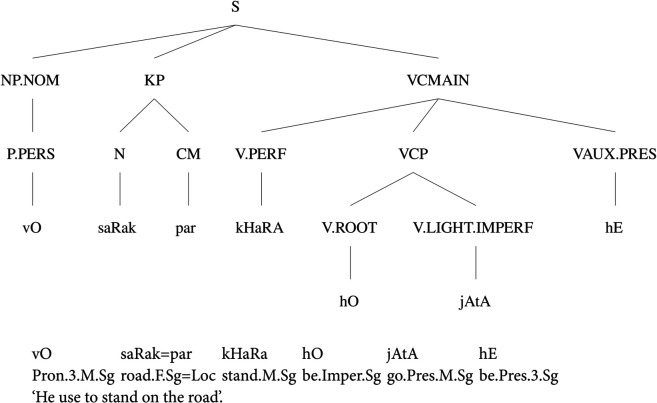
A sample PS parse tree from the Urdu.KON-TB.

The Urdu.KON-TB has multiple annotation layers, including POS tags, phrase labels, and functional tags. The phrase labels have morphological information attached with them. The phrase label concatenates the information of tense, case, aspect, and modality. The treebank has been annotated using a large annotation scheme containing 26 phrase labels. However, the treebank contains only 1,400 sentences with an average of 13.73 tokens per sentence. The corpus has been collected mainly from the Urdu Wikipedia (https://ur.wikipedia.org) and the Jang newspaper (https://jang.com.pk). Fig 1 demonstrates a sample annotated sentence from the Urdu.KON-TB. The Urdu text in all examples has been written using a transliteration scheme proposed in [48].

The parse tree in Fig 1 shows the attachment of three clauses under the ‘S’ phrase label. The ‘S’ label depicts the annotation of the whole sentence that has an NP.NOM, a KP, and a VCMAIN. The NP.NOM defines the nominal case along with a noun phrase. The POS tag for personal pronoun is P.PERS which marks the pronoun by depicting its type as well. The nominal case represents the nominal subject. The second clause annotates a case marking clause with the KP label. The annotation of the case phrase is quite flat as noun and case marker appear at the same level. The third clause defines the verbal structure with the VCMAIN label. It contains a main verb annotated with V.PERF that shows the verb and its tense, a VCP clause that contains the root of the verb and a light verb, and finally an auxiliary verb by using a VAUX label along with the tense information. The verbal construction is quite complicated as it demonstrates a main verb *kHaRA* ‘stand.M.Sg’ with a root *hO* ‘be.Imper.Sg’ and a light verb *jAtA* ‘go.Pres.M.Sg’. The verbal construction actually makes a complex predication to denote adjective+verb construction. This annotation lacks annotation of different order of arguments within a complex predicate. Nouns or adjectives do not always appear with verbs; therefore, a different annotation approach is required that has the ability to accommodate the flexible word-order of the language. The Urdu.KON-TB divides the verbal structure into four categories that include a VCMAIN; which annotates the main clause, a VCP; to annotate complex predicates, a VIP; that marks infinitive verb phrase, and a VP for simple verb phrases. The Urdu.KON-TB has a large annotation label set that contains the duplicate labels for questions like NPQ, KPQ, QWP, ADJPQ, ADVPQ, SBARQ and SQ.

The linguistically rich annotation scheme of the Urdu.KON-TB provides a deeper linguistic insight into the language. However, it is a small treebank that has been annotated using a complex labeling scheme. Although statistical and neural parsing models require large amounts of data for better performance, Urdu.KON-TB has been analyzed to perform dependency parsing by converting it to dependency structure in [49]. The well-known MaltParser [50] was used to perform dependency parsing. Despite the richer annotation scheme, the conversion to dependency structure was incorrect due to the deeper hierarchical structure of the parse trees. Based on experiments, the authors concluded that the Urdu.KON-TB is not suitable for dependency parsing.

**Fig 2:**
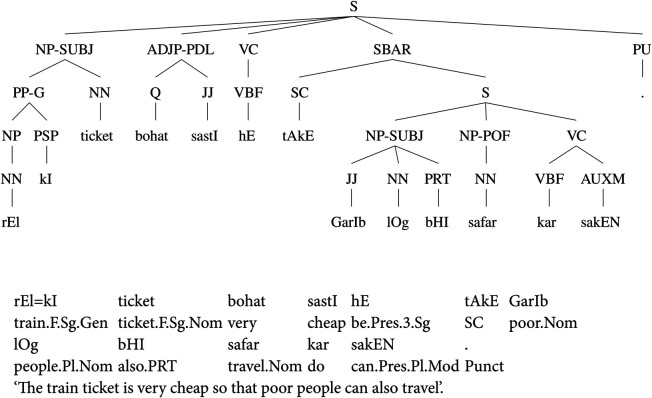
A sample PS parse tree from the CLE-UTB.

On the other hand, the CLE-UTB has a relatively flat annotation structure. The flat structure is helpful for annotating the flexible word order of the language by attaching arguments in any order in a parse tree that is a key attribute of the dependency structure. The annotation scheme of the CLE-UTB has the ability to represent frequently observed syntactic constructions of the language, including flexible word-order, complex predicate structure, question phrases, subordination, conjunctions, genitives, and relative clauses. Additionally, the CLE-UTB has a set of functional labels to mark grammatical relations. Fig 2 presents a sample annotated sentence from the CLE-UTB.

The PS parse tree in Fig 2 shows the annotation of a genitive case and a copula construction in the independent clause. The genitive case is annotated by using a post-positional phrase (PP) and a function label ‘G’. The PP-G phrase is a component of the nominal subject that has *ticket* as the head word. The independent clause further annotates a copula construction that marks an adjective phrase (ADJP) with a functional label PDL (Predicate Link). The PDL construction makes a link with the predicate, which, in this case, is a copula verb *hE* ‘be.Pres.3.Sg’.

The subordinate clause, on the other hand, contains a nominal subject and a complex verbal structure. The NP-SUBJ phrase contains an intensifier *bHI* ‘also’ in it. The nominal subject is followed by a complex predicate structure that has been marked by using a Part-of-Function (POF) label. The noun phrase along with POF provides a verbal meaning generally followed by a Verbal Complex (VC). In the complex predicate structure, the predicate contains a light verb, *kar* ‘do’ in this case, which is further followed by a modal auxiliary verb *sakEN* ‘can.Pres.Pl.Mod’.

It is important to note that all the arguments and predicates are attached at the clause level in the parse tree shown in Fig 2. This annotation scheme of the CLE-UTB allows the annotation of sentences having different word orders. The functional layer makes the annotation flexible, and predicates can be marked irrespective of their order in the sentence. Similarly, copula constructions can be annotated independently of their position. Predicates and their arguments including subjects, objects, obliques, conjuncts, infinitive clauses, subordinate and relative clauses are annotated without restricting them to have specific positions in a parse tree.

The annotation scheme of the CLE-UTB is flat and has fewer labels compared to the Urdu.KON-TB. The CLE-UTB’s labels including POS tags, phrase and functional labels are also compatible with existing resources like the well-known Penn Treebank. However, some POS tags and phrase labels have been added to represent the grammatical structure of Urdu. The flat annotation approach makes it compatible with the dependency structure [16,17]. The CLE-UTB is sufficiently large that has 7,854 sentences having text from fifteen different genres. In this work, we have transformed the CLE-UTB from phrase structure to dependency structure and performed multi-task learning-based parsing.

In recent years, Urdu NLP has witnessed significant advancements in a variety of core tasks. In semantic parsing, the first semantically annotated meaning bank for Urdu was created to enable neural parsing and generation, leveraging cross-lingual alignment and extensive data augmentation yielded a major boost in parsing accuracy [51]. In machine translation, systematic benchmarks show that specialized multilingual models can outperform general-purpose LLMs for English-Urdu translation. For example, the IndicTrans2 model achieved the highest translation quality on multiple test sets, significantly outperforming both a GPT-3.5-based translation model and a conventional bilingual Transformer [52]. A recent study shows that it is crucial to use optimized part-of-speech (POS) tagging to improve the performance of Urdu text classification. A new POS tagging technique, coupled with SVM variants and ensemble models, led to significant improvements in classification accuracy, achieving over 98% average scores on several evaluation measures on a four-domain corpus [53]. Another recent research explored the use of Large Language Models (LLMs) to address dynamic label problems in large-scale multi-label text classification (LMTC). A recent strategy focused on DyLas (Dynamic Label Alignment Strategy) applies counterfactual analysis to align labels without the requirement of retraining a model and demonstrated performance improvements with different datasets and open and closed source LLMs [54].

Low-resource challenges are also being addressed by new resources for higher-level tasks such as summarization and question answering. The first multimodal Urdu summarization dataset (UrduMASD) was introduced in 2024 to enable abstractive summaries from videos and text [55]. Similarly, a large-scale QA corpus (UQA) was constructed by translating the SQuAD2.0 benchmark [56] into Urdu using a span-preserving method [57], and state-of-the-art multilingual transformer models have already achieved strong performance on this dataset. Conversational generation has made strides recently in overcoming the basic issues of semantic ambiguity and contextual coherence through better transformer-based models. A novel approach combining pre-trained word embeddings with character information and sparsity-regularized Softmax loss resulted in substantial improvement in BLEU and distinct scores with better coherent and contextually relevant responses on the STC dataset [58].

Previous work has extended clickbait detection research to the low-resource Urdu language, which was not addressed in this task previously. Using sentence embeddings with deep learning methods, researchers achieved 88% accuracy in identifying clickbait headlines, much higher than typical machine learning baselines [59]. The advent of large language models is beginning to benefit Urdu. Researchers have introduced UrduLLaMA 1.0, an 8-billion-parameter model tailored to Urdu by continual pretraining and LoRA fine-tuning on Urdu data, which has set new benchmarks on several Urdu NLP tasks by substantially improving prior state-of-the-art results [60].

In the field of speech processing, recent multilingual approaches have greatly improved Urdu speech recognition and synthesis. On the recognition side, state-of-the-art models (e.g. OpenAI Whisper, Meta’s MMS and SeamlessM4T) have been fine-tuned and benchmarked on Urdu, aided by the release of the first large-scale Urdu conversational speech dataset [61]. These evaluations show that fine-tuned models can achieve relatively low word error rates for Urdu (whisper performing best in conversational speech), although challenges such as accent variability and the need for robust text normalization persist [61].

In text-to-speech, the lack of Urdu training data is being addressed by transfer learning: for instance, a study constructed an Urdu speech corpus from audio books and then used a pre-trained neural Text-to-Speech (TTS) model (trained on a high-resource language) to generate intelligible Urdu speech [62]. The resulting system demonstrated satisfactory naturalness despite the limited data, although there is still room for improvement. Meanwhile, Urdu conversational agents have begun to emerge in real-world applications. A notable example is the “SAATHI" virtual assistant, which provides an intuitive Urdu voice interface for elderly users, helping with daily tasks such as reminders of medications, information access, and even offering empathetic conversations for companionship [63]. Recent contributions to Urdu speech processing have introduced a TTS system powered by Deep Learning techniques using Tacotron 2 with WaveGlow to generate natural-sounding speech from Urdu text. The system achieved a Mean Opinion Score (MOS) of 3.76, overcoming previous research on Urdu TTS that lacked advanced neural architectures [64]. Such developments illustrate how advances in language resources and models are making Urdu NLP technologies more robust and accessible across both text and speech domains.

## 3 PS to DS conversion

We conducted the conversion process in three steps. In the first step, we generated an intermediate dependency representation by implementing a head-word model. This representation contains dependency arcs along with phrase and POS tags. In the second step, we performed the phrase-to-dependency label mapping to transform the intermediate representation into the dependency structure. Finally, in the third step, we applied post-conversion rules to generate the final dependency treebank. This section outlines the phrase-to-dependency structure conversion by detailing the Urdu head-word model, phrase-to-dependency label mapping, post-conversion rules, and the conversion evaluation.

### 3.1 Urdu head-word model

In DS, a head-word is marked with the core dependency labels. A head-word is a dominant lexical item in a phrase. A head-word algorithm finds head-words from clauses by implementing language-dependent rules [65]. We have proposed a head-word model for the CLE-UTB treebank. In our treebank conversion process, we refined an existing algorithm (https://github.com/Luolc/CTB2Dep) [66]. Table 1 shows label-wise details of the proposed head-word model.

**Table 1:** Head-word model for the CLE-UTB treebank.

Phrase labels	Direction	Priority
VC	left	VBF, VBI, AUXA, AUXM, AUXP, AUXT, VC, NEG
PP	left	NP, S, QP, NNP, NN, PP, PSP
NP	right	NP, NNP, NN, PRP, PRR, S
ADJP	right	ADJP, JJ, Q, QP, RB
QP	right	QP, Q, CD, OD, FR, QM, JJ
ADVP	right	ADVP, RB, NP, NN
PREP	right	NP, NNP, NNP, PREP
DMP	right	PDM, PRP, PRT
FFP	left	FF, NNP, NN
S	left	VC, S, SBAR, NP, ADJP, QP, NNP, NN, PRP
SBAR	left	S, SBAR, SCK

Table 1 presents the word finding direction and the priority list for each phrase label. A verb complex generally contains a main verb followed by one or more auxiliary verbs. Therefore, the head-finding direction is from left to right with respect to the priority of labels. A VC mostly has finite verbs as main verbs; therefore, the POS tags VBF and VBI have high priorities compared to the auxiliaries. The verbal constituent in (1) demonstrates a typical Urdu verbal structure which contains the main verb *likH* ‘write’, a light verb *liyA* ‘take.Perf.M.Sg’ and a tense auxiliary verb *hE* ‘be.Pres.3.Sg’. The main verb should be the head of the phrase and have the core dependency label that appears on the left-hand side. It is important to note that Urdu is written from right-to-left, but its internal representation is a sequence of left-to-right Unicodes, therefore, the directions in our head-word model have been devised for internal representations of the writing script.

(1) likH liyA hE
   write.Imperf.Sg take.Perf.M.Sg be.Pres.3.Sg
   ‘has written’

Similarly, post-positional phrases (PPs) have left heads as they contain at least one noun phrase that appears before a case marker. Therefore, the PP has NP to have the highest priority followed by other clause labels. In (2), a PP sequence contains a noun phrase *surx mEz* ‘red table’ followed by a locative case marker *par* ‘on’. The head-word of the noun phrase becomes the head of the PP phrase. The general direction of the head of the PP is from left-to-right. Besides NPs, a PP may have other phrases that have been added to its priority list.

(2) surx mEz=par
   red.Sg table.M.Sg=Loc
   ‘on the red table’

The noun phrase shown in (3) contains a genitive case, and hence an internal post-positional phrase. The head of the NP is the word *pensil* ‘pencil’ which appears on the right-hand side of the phrase. Similarly, the NP sequence presented in (4) shows a canonical NP construction that contains the noun *Am* ‘mango’ as head-word after a noun modifier *mItHA* ‘sweet’. The head-finding direction of noun phrases is from right-to-left. A noun phrase can also have internal NPs, common and proper nouns and pronouns that all are right headed.

(3) sAim=kI pensil
   Saim.M.Sg=Gen pencil.F.Sg.Nom
   ‘Saim’s pencil’
(4) mItHA Am
   sweet.Sg mango.M.Sg.Nom
   ‘sweet mango’

The noun modifiers also have right heads as shown in (5) and (6). An adjective phrase, in the annotation, can have quantifiers, cardinals, ordinals, and main adjectives. The order of an adjective phrase is similar to that of canonical NPs. The head modifier appears on the right-hand side of the constituent. In the same way, the quantifier phrase also has right heads, as shown in (6).

(5) buhat mazbUt
   very strong.M.Sg.Adj
   ‘very strong’
(6) buhat kam
   very less.M.Sg.Adj
   ‘very less’

An adverbial phrase (ADVP) mainly contains a single lexical entry for an adverb as presented in (7). In some cases, the ADVP contains an infinitive clause, but the head-word remains on the right-hand side of the constituent.

(7) acAnak
   suddenly.Adv
   ‘suddenly’

Urdu also has prepositional phrases, but they are not frequent in corpora. A prepositional phrase contains an NP that follows the preposition clitic, which makes it right headed as shown in (8). The preposition clitic *fI* ‘per’ appears before a noun phrase making the noun the head of the constituent.

(8) fI gHanTA
   per hour.M.Sg.Nom
   ‘per hour’

**Fig 3:**
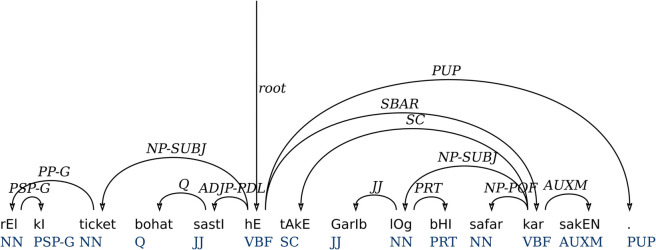
An intermediate dependency representation from PS parse tree of Fig 2 after head identification.

A demonstrative phrase (DMP) contains a demonstrative pronoun followed by a particle, making its head-finding method straight-forward from right-to-left. Similarly, foreign fragment phrase (FFP) annotates the foreign language text segments, generally from English and Arabic in the treebank. All foreign words are assigned the POS tag of FF. We assume it to be a left-headed constituent. The ‘S’ label is used to annotate clauses or a whole sentence. A sentence can have subordinate and coordinate clauses in it. The SBAR label is used to annotate subordinate clauses with the label ‘S’. The clause or sentence has the main verb as the head of the sentence. Therefore, it looks for a left head of the matrix clause as the head of the whole sentence. In Fig 3, a hierarchical analysis and parental annotation have been performed to determine heads for clause ‘S’. The parental annotation was helpful to achieve the context and phrase-to-dependency label mappings.

Fig 3 shows an intermediate dependency representation of the sentence in Fig 2. The representation is achieved by finding dependency arcs for head-words. The remaining items of the constituents show dependency arcs on the respective heads. This representation has phrase and functional labels as relations. Besides labels, the dependency arcs are correct with respect to the Universal Dependencies. The construction including subordinate clause, subjects, complex predicate, genitive case, and auxiliary verbs show dependency arcs on their respective head-words.

### 3.2 PS to DS label mappings

The intermediate dependency representation contains dependency arcs for head and non-head tokens. The non-head-words have POS tags as arc labels and heads have phrase labels as dependency relations. During the conversion process, we designed a refined version of the POS tag set that divides case markers and punctuation marks into multiple categories according to their syntax. The POS tag set originally had 35 tags, which are now extended to 44 tags. The Universal Dependencies (UD) V-2.0 label set has been used for dependency labels and POS tags. The universal POS tag set contains 17 tags (https://universaldependencies.org/u/pos). In addition to the UD guidelines, the Hindi-Urdu Treebank (HUTB) [67] has been analyzed for label mappings and post-conversion rules.

In Fig 3, the noun *ticket* is the head-word and shows a dependency relation with the NP-SUBJ label, which makes it intuitive that it is a subject. The noun *rEl* ‘train’ shows a dependency on the head with the PP-G label. The PP-G label is used to annotate post-positional phrases for genitive case markers (clitics). The Urdu script treats case marking clitics as independent tokens hence requires a dependency label. The genitive case marker *kI* has a dependency on the noun *rEl* which is a possessor in the sentence. The quantifier *bohat* ‘very’ has a dependency on the adjective *sastI* ‘cheap’ with the ‘Q’ label which is a POS tag. The adjective *sastI* is the head-word with the core dependency label ADJP-PDL. Its appearance before a copula verb *hE* makes a construction called a predicate link. We updated such copula constructions by implementing post-conversion rules.

The subordinate clause contains a subject and a complex predicate structure. The phrase labels NP-SUBJ and NP-POF, representing dependency relations for heads and POS tags, are labeled to show non-core relations. For example, the noun modifier *GarIb* ‘poor’ has a dependency on the noun *lOg* ‘people’ with the JJ label. The modal auxiliary is defined by the AUXM tag. The particle *bHI* ‘also’ is labeled with the PRT POS tag. The subordinate clause is marked with the SBAR label. It is important to note that phrase labels along with functional labels represent dependency relations of heads on the predicates, and POS tags depict relations of non-head items on constituent heads. Therefore, to generate dependency labels, the mapping to dependency labels should be performed for both phrase labels and POS tags.

To achieve the context of constituents for accurate label mappings, we updated CLE-UTB to have parental annotations [68]. A clause ‘S’ may have different attachments that can be identified by using the parent label annotations along with its actual label. For example, if the clause ‘S’ appears within a noun phrase, it is annotated as S^NP and if it is attached with a post-positional phrase, it would have the annotation as S^PP. The subordinate and coordinate clauses are also annotated with an ‘S’ label that show S^SBAR for a subordinate clause and S^S for a coordinate clause. In addition to the parental annotation, tree levels are also annotated for VCs that are used to identify the roots of sentences. A sentence with many clauses has many verbal constructions in a hierarchical parse tree. The annotation of the level number attaches a number, for example, VC^1 represents the verbal construction at the first level, considering the sentence clause at the zeroth level.

#### 3.2.1 Core arguments.

Table 2 presents the mapping of phrase labels for core arguments to UD labels. The functional labels represent the core arguments for subject and object. The subordinate and coordinate clauses are marked with the ccomp (clausal complement) label. Parental annotations have been used to identify clauses. The nominal obliques have been mapped on indirect objects iobj. This mapping was further refined by employing the second rule described in Sect 3.3. Infinitive clausal objects have been marked as indirect objects with the xcomp label with respect to the HUTB. The labels with a ‘*’ are achieved by additional rules described in Sect 3.3.

**Table 2:** The mapping of the CLE-UTB phrase labels on UD labels for core arguments.

CLE-UTB lables	UD-2.0 labels	UD description
NP-SUBJ	nsubj	Nominal subject
PP-SUBJ	nsubj	Nominal subject
QP-SUBJ	nsubj	Nominal subject
NP-OBJ	obj	Object
PP-OBJ	obj	Object
QP-OBJ	obj	Object
S-OBJ	obj	Object
NP-OBL^*^	iobj	Indirect object
S-SUBJ	csubj	Clausal subject
SS	ccomp	Clausal complement
SBAR	ccomp	Clausal complement
SSBAR	ccomp	Clausal complement
S^*^	xcomp	Open clausal complement

* These dependency labels are achieved by post-conversion rules.

#### 3.2.2 Non-core dependents.

Table 3 presents the mapping of non-core dependencies. The non-core compulsory arguments are annotated by using the OBL functional label in the CLE-UTB that is mapped on the oblique dependency with the obl tag. The functional label VOC has been mapped on vocative label. Non-core finite clauses are marked using the advel (adverbial clause modifier) label. Similarly, clauses with conjunctive participle are also marked with advel. These types of clauses are attached under the SBAR label that are identified by using parental annotations. Adverbial phrases (ADVP) and nominal adjuncts have been mapped on adverbial modifier dependency relation using the advmod label. The noun phrase, post-positional phrase, prepositional phrase, and quantifier phrase that do not have any functional label are considered adjuncts and are marked with an adverbial modifier. The POS tags RB and NEG for adverbs and negative adverbs, respectively, are also mapped on the advmod label.

**Table 3:** The mapping of the CLE-UTB phrase labels on UD labels for non-core dependents.

CLE-UTB lables	UD-2.0 labels	UD description
NP-OBL	obl	Oblique nominal
PP-OBL	obl	Oblique nominal
S-OBL	obl	Oblique nominal
ADVP-VOC	vocative	Vocative
NP-VOC	vocative	Vocative
S	advcl	Adverbial clause modifier
SSBARSCK	advcl	Adverbial clause modifier
SBARSCK	advcl	Adverbial clause modifier
ADVP	advmod	Adverbial modifier
NP	advmod	Adverbial modifier
NP-ADJ	advmod	Adverbial modifier
PP	advmod	Adverbial modifier
PREP	advmod	Adverbial modifier
QP	advmod	Adverbial modifier
NEG	advmod	Adverbial modifier
RB	advmod	Adverbial modifier
ADVP-INJ	discourse	Discourse element
INJ	discourse	Discourse element
AUXA	aux	Auxiliary
AUXM	aux	Auxiliary
AUXP	aux	Auxiliary
AUXT	aux	Auxiliary
SCK	aux	Auxiliary
ADJP-PDL	cop	Copula
ADVP-PDL	cop	Copula
NP-PDL	cop	Copula
PP-PDL	cop	Copula
QP-PDL	cop	Copula
SC	mark	Marker
SCP	mark	Marker

Adverbial interjections are marked with the discourse label. All types of auxiliary verbs and conjunctive participles are mapped on aux label. The annotation of the CLE-UTB utilizes a PDL functional label to annotate predicate links with copula verbs; therefore, constructions with PDL labels are mapped on copula dependency with the cop label. The labels for subordinate clitics are mapped on the mark dependency label.

#### 3.2.3 Nominal dependents.

Table 4 presents the mapping of the nominal dependents. Genitive cases are marked as noun specifiers and possessors, and are annotated with the PP-G phrase in the PS treebank. For conversion, PP-G is marked as a nominal modifier using the nmod label. Cardinals are marked as numeric modifier by using the nummod label. The adjectival phrases and adjectival tags are mapped as adjectival modifiers. The adjectival clauses that are identified through parental annotations in the ‘S’ clause, are mapped on the acl label. The determiner label det is used to represent demonstrative phrases, APNA particles, fractions, ordinals, quantifiers, and multiplicatives. Different types of pronouns, including demonstrative, relative demonstrative, reflexive, relative, and possessive, are also marked as determiners. The dependency label case is used to map the annotation of all types of case markers in the CLE-UTB.

**Table 4:** The mapping of the CLE-UTB phrase labels on UD labels for nominal dependents.

CLE-UTB lables	UD-2.0 labels	UD Description
PP-G	nmod	Nominal modifier
CD	nummod	Numeric modifier
ADJP	amod	Adjectival modifier
JJ	amod	Adjectival modifier
S^ADJP	acl	Adjectival clause
S^NP	acl	Adjectival clause
S^PP	acl	Adjectival clause
DMP	det	Determiner
APNA	det	Determiner
FR	det	Determiner
OD	det	Determiner
PDM	det	Determiner
PRD	det	Determiner
PRF	det	Determiner
PRP	det	Determiner
PRR	det	Determiner
PRS	det	Determiner
Q	det	Determiner
QM	det	Determiner
PRE	case	Case marking
PSP	case	Case marking
PSP-G	case	Case marking
PSP-I	case	Case marking
PSP-A	case	Case marking
PSP-E	case	Case marking
PSP-SE	case	Case marking

#### 3.2.4 Other dependency relations.

Table 5 presents the dependency labeling for coordination, multi-word expressions, punctuation, roots, and other unspecified dependencies. The conjunctions within noun, adjective, and quantifier phrases that are separated with commas are identified on the bases of POS tags and are marked with the conj label. The coordinate conjunction tag CC is mapped on the cc dependency label. The noun, adjective, and quantifier phrases associated with the internal case marker PSP-I are mapped on the fixed label. The foreign fragment phrases mostly have a flat structure, therefore they are mapped on the flat dependency label. The compound nouns with more than one lexical items have rightmost word as a head-word and the non-head lexical items are mapped on the flat label. An example of such noun phrases is *mOm battI* ‘candle’. In Urdu, both *mOm* ‘wax’ and *battI* ‘light’ are nouns. In the head-word model, the noun *battI* is the head and *mOm* has a flat dependency relation on the head.

**Table 5:** The mapping of the CLE-UTB phrase labels on UD labels for dependency relations.

CLE-UTB lables	UD-2.0 labels	UD description
NP^*^	conj	Conjunct
NN^*^	conj	Conjunct
ADJP^*^	conj	Conjunct
JJ^*^	conj	Conjunct
QP^*^	conj	Conjunct
Q^*^	conj	Conjunct
CC	cc	Coordinating conjunction
NP^*^	fixed	Fixed multiword expression
ADJP^*^	fixed	Fixed multiword expression
QP^*^	fixed	Fixed multiword expression
FF	flat	Flat multiword expression
NN	flat	Flat multiword expression
NP-POF	compound	Compound
ADJP-POF	compound	Compound
QP-POF	compound	Compound
VC-VALA	compound	Compound
LRB	punct	Punctuation
PU	punct	Punctuation
PU-C	punct	Punctuation
PU-E	punct	Punctuation
PU-P	punct	Punctuation
RRB	punct	Punctuation
VC	root	Root
FFP	dep	Unspecified dependency
PRT	dep	Unspecified dependency

* These dependency labels are achieved by using post-conversion rules.

The annotation of complex predicate structures in the treebank is represented by using the POF functional label. The functional label is generally attached with noun, adjective, and quantifier phrases. In the conversion process, phrases with POF label are mapped on the compound label. The verbal construction with *vAlA* particles are also marked as compounds. All types of punctuation marks are mapped on the punct label. The heads of the verbal constructions are marked as root. The verbal structure of the main clause at the highest level is marked as root. The dep label is used to mark unspecified dependencies. The items in foreign fragment phrases have flat dependencies on heads, but their heads have an arc to the root of the sentence. We have marked such relations as unspecified dependencies. Similarly, particles are used as intensifiers and therefore marked with the dep label during conversion.

### 3.3 Post-conversion rules

For most of the phrase labels, dependency mapping is quite straight-forward as the phrase annotation scheme has functional labels that represent grammatical relations. Due to the flat annotation of the CLE-UTB, complex structures were mapped by implementing several post-conversion rules to generate an accurate DS treebank. The first mapping issue is identified for the conversion of indirect objects. The annotation of the CLE-UTB does not annotate secondary objects but marks them as obliques by attaching OBL functional label. However, Urdu uses the accusative case marker *kO* for secondary objects and recipients. Similarly, non-finite clauses are annotated similar to finite clauses. These clauses are identified by their verbal heads. Furthermore, rules are derived for fixed and conj labels. Various rules are devised to make the generated treebank compatible with the Hindi-Urdu Treebank [67]. The details of the conversion rules are as follows.

1. One way to represent secondary objects and recipients is the use of accusative/dative case marker *kO* in Urdu. The annotation of the CLE-UTB annotates all case marking constructions with post-positional phrase (PP). The annotation marks secondary objects as obliques hence with the label PP-OBL. So the rule is that, if the label is the PP-OBL and the next lexical item is a clitic *kO*, then map it on the iobj dependency label.
2. There are nominal secondary objects that are annotated as nominal obliques. These constructions include certain types of pronouns to provide meanings of recipients or beneficiaries. A list of such pronouns with their meanings is shown in Table 6. If the phrase label is NP-OBL and the head of the phrase is any of these pronouns, then mark the dependency arc with the iobj label.
3. The CLE-UTB annotates non-finite clauses and clausal objects similar to other clauses by using the ‘S’ and S-OBJ labels. We map such constructions on the xcomp dependency label. If a construction has any of these labels and their phrasal head is an infinitive verb (having the VBI POS tag), then map the construction with the xcomp dependency label.
4. Some constructions contain an internal clitic as a connection between two nouns, adjectives, and quantifiers. For instance, the constituent *kam-az-kam* ‘at least’ has a clitic *az* ‘from’ in it. The clitic is tagged with the PSP-I POS tag. The constructions with the PSP-I tag are mapped on the fixed dependency label.
5. The conjunctions between nouns, adjectives, and quantifiers are represented by using conjunction clitics and/or commas. A comma is used when there are more than two lexical items. We map such constructions on the conj label if they use conjunctions with the CC or comma with the PU-C POS tag.
6. The HUTB annotates case markers that follow infinitive verbs, in a different manner. Such case markers are tagged with the mark label rather than case. We find such case markers and labeled them with the mark dependency label to make our generated dependency treebank compatible with the HUTB.
7. The CLE-UTB annotated spacio-temporal nouns similar to common nouns. However, the HUTB uses the NST POS tag for such nouns and annotated them with the case dependency label. Therefore, we compiled a complete list of such nouns from the HUTB and updated their labeling in the conversion process. If an NST noun appears after a case marker, then it is annotated with case label and its head is the head of the preceding case marker.
8. If two case markers appear together, then the head of next case marker is also the head of the previous case marker and they both are annotated with the case dependency label.
9. The CLE-UTB annotates the spacio-temporal nouns by using a functional label ADJ. If a noun is not in NST list but has NP-ADJ as the original label, then it is assigned the obl dependency label. If a token has NP-ADJ label but it is present in the NST list and the previous token is not a case marker, then it is marked as adverbial modifier by using the advmod label.

**Table 6:** List of pronouns marked as indirect objects.

Sr.#	Pronouns	Romanized	Translation	Grammatical function
1	مجھے	*mujHE*	To/for me	1st person singular
2	ہمیں	*hamEN*	To/for us	1st person plural
3	تجھے	*tujHE*	To/for you	2nd person informal
4	تمہیں	*tumEN*	To/for you	2nd person polite/plural
5	اِسے	*isE*	To/for him/her	Near, 3rd person singular
6	اُسے	*usE*	To/for him/her	Far, 3rd person singular
7	اِنہیں	*inhEN*	To/for them	Near, 3rd person plural
8	اُنہیں	*unhEN*	To/for them	Far, 3rd person plural
9	جسے	*jisE*	The one who	Relative pronoun, singular
10	جنہیں	*jinhEN*	To/for those who	Relative pronoun, plural
11	کسے	*kisE*	To/for whom	Interrogative pronoun

**Fig 4:**
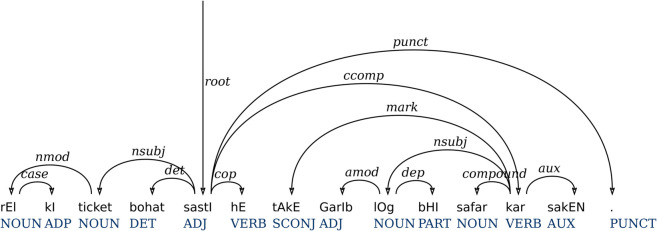
Final dependency tree from the intermediate tree of Fig 3 after label mappings and post-conversion rules.

Fig 4 presents the final dependency tree of the intermediate structure after applying label mappings and post-conversion rules. The dependency arcs in Fig 4 are quite similar to the arcs in Fig 3 except for the copula construction. All phrase labels are replaced by the dependency labels. The copula construction is updated with respect to dependency labels. The predicate link is marked as root of the sentence, and the copula verb *hE* has a cop dependency on the root. The generated treebank has 27 unique dependency labels that are given in Table 7.

**Table 7:** UD labels that are used by generated dependency treebank.

Universal dependency labels			
acl	advcl	advmod	amod
aux	case	cc	compound
conj	cop	csubj	dep
det	discourse	fixed	flat
iobj	mark	nmod	nsubj
nummod	obj	obl	punct
root	vocative	xcomp	—

### 3.4 Conversion evaluation

The evaluation is a mandatory task during the development of a computational data resource. Inter-annotator agreement scores are computed to represent the understanding of the annotators during the annotation process. In our case, the PS treebank has already been evaluated using different methods. There was no reference corpus available to compute similarity or reference scoring for the generated dependency treebank. For this purpose, we obtained an existing dependency treebank from the Hindi-Urdu treebank project [67]. The project contains separate repositories for Hindi and Urdu. Each repository has training, test, and development sets. We took the first hundred sentences from the development set of the Urdu treebank repository and manually annotated them using the annotation guidelines of the CLE-UTB presented in [69]. This reference corpus was then converted to the dependency structure and compared with its original annotation. This method may not completely evaluate the conversion process but provides an insight of the agreements for core arguments of the language.

The reference corpus has been evaluated by computing the labeled attachment score (LAS), the unlabeled attachment score (UAS), the label accuracy (LA), and Cohen’s Kappa coefficient [70]. Table 8 shows the details of the evaluation scores.

**Table 8:** Inter-annotator agreement scores on reference corpus containing 100 sentences.

Category	UAS	LAS	LA
Dependency measures	79.21	69.59	80.62
Kappa Coefficients	0.7859	0.6942	0.7857

Evaluations are performed on the basis of tokens, their labels, and heads. The unlabeled attachment score shows the agreement of heads irrespective of their labels. However, the labeled attachment score shows the accuracy where both labels and heads are correct. The label accuracy shows the percentage of correct labels. Cohen’s kappa coefficient computes the inter-rater agreement and its interpretation is shown in the Table 9. The agreement for heads and labels is quite acceptable as it is almost 0.80 showing strong agreement. However, the score for labeled attachment is near 0.70 which represents moderate agreement.

**Table 9:** Interpretation of Cohen’s Kappa coefficient values.

Kappa value	Agreement level
0 - 0.20	None
0.21 - 0.39	Minimal
0.40 - 0.59	Weak
0.60 - 0.79	Moderate
0.80 - 0.90	Strong
0.91 and above	Almost Perfect

The annotation of both treebanks is different as the Urdu treebank from the HUTB project has been annotated manually. On the other hand, our dependency treebank is generated from a PS treebank by utilizing functional labels to represent dependency relations. These labels are limited to represent all types of dependency relations. However, the generated dependency has the representation of major types of core and non-core arguments.

## 4 Sequential representations

The parsing task can be simplified by transforming the hierarchical representation into sequence labeling. Both phrase and dependency structures can be converted to sequence labels and back to the hierarchical structure accurately. Sects 4.1 and 4.2 present the transformation of PS and DS trees into sequence labels.

### 4.1 PS tree representation

**Fig 5:**
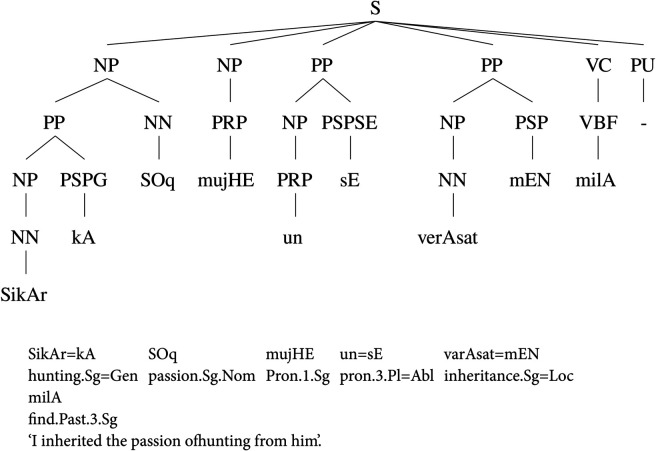
A sample PS parse tree along with sequence labels.

For the conversion of PS trees to sequential format, we use a baseline transformation approach as discussed in [71]. The label output is in CoNLL format containing tokens, POS tags, and tree labels. The treebank labels consist of an integer and a tag. The integer, which is part of a label, represents the total number of common ancestors. The phrase label, attached with the integer, represents the common label between successive tokens at the lowest level in the parse tree. However, unary branches are handled separately by attaching them with POS tags. Fig 5 shows an Urdu PS parse tree followed by the proposed linear labeling (9) compared to the relative and absolute labeling presented in [71].

(9) Tokens: SikAr kA SOq mujHE un sE varAsat mEN milA
   Relative [71]: 3_PP -1_NP -1_S 0_S 1_PP -1_S 1_PP -1_S 0_S
   Absolute [71]: 3_PP 2_NP 1_S 1_S 2_PP 1_S 2_PP 1_S 1_S
   Proposed(our’s): PP 2_NP 1_S 1_S PP 1_S PP 1_S 1_S
   -
   NONE
   NONE
   NONE
   ‘I inherited the passion of hunting from him’.

A simple (absolute) linear encoding produces a label using the common number of phrase labels between *w*_*i*_ and $w_{i}\;+\;1$. However, it may generate a large number of labels. The second encoding method proposed by [71] uses the difference of common ancestors from the next $w_{t}\;+\;1$ and previous token $w_{t}\;-\;1$. For example, the token *kA* (genitive case marker) has a relative scale label −1_NP. The token *kA* has two common ancestors, NP and S, with the next token *SOq* ‘passion’ and three common ancestors, PP, NP and S, with the previous token *SikAr* ‘hunting’. Therefore, the difference is −1 and the lowest common ancestor label with the next token is NP which produces the linear label −1_NP. This method is referred as relative scale and generates remarkably less number of labels as compared to absolute scale encoding. The unary branch labels are produced separately at the first step of the labeling that are further combined with POS tags for the purpose of conversion back to brackets (phrase structure). Therefore, the experiments are performed in two passes. The first pass predicts unary labels and the second pass learns the relative phrase labels. The output is converted back to parse trees to perform evaluations.

The analysis and experiments of relative and absolute labeling were performed for the English Penn Treebank. The annotation of the CLE-UTB has a relatively flat structure hence with less number of labels. We propose a labeling scheme that has the ability to generate even fewer labels. The average entropy of our proposed labeling is also lower than the relative labeling. The proposed label set produces better parsing scores for the CLE-UTB and outperformed the relative labeling scheme. Table 10 presents the comparison of both labeling schemes.

**Table 10:** Comparison of the proposed sequential labeling with the labeling of [71] with respect to the CLE-UTB.

Sequence labeling	# of labels	Entropy (average)
Relative [71]	180	3.15
Proposed(our’s)	143	2.65

Table 10 shows the number of labels and the average entropy for the entire treebank using relative and proposed labeling schemes. The entropy values depict the randomness of labels for a sentence. *X* is the random variable given that has possible outcomes $x_{1},x_{2},...,x_{n}$ with probability $P(x_{1}),P(x_{2}),...,P(x_{n})$, the entropy of the variable *X* is represented by Eq 1.

$$H\left(X\right)=-\sum_{i=1}^{n}P\left(x_{i}\right)\log P\left(x_{i}\right)$$ (1)

The entropy value of the proposed labeling is less than the relative labeling, showing that the proposed labeling has less randomness and hence has a higher learning capability in the training process. The proposed labeling is based on the absolute labeling approach that labels a common number of phrase labels of a token and its next token. The label further concatenates the lowest common phrase with it. Similarly to relative labeling, a label consists of a number followed by a phrase label.

Absolute labels were analyzed against the annotation of the CLE-UTB to achieve predictable patterns. There were several patterns due to the simplified and flatter syntactic structure of the PS treebank. We analyzed them to reduce the number of labels. The number of common phrase labels in a PP phrase is always one higher than the number of next labels. In absolute labeling, the first label is 3_PP and the second label is 2_NP. Similarly, fifth and seventh tokens have 2_PP label and their next label is 1_S. This sequence is found throughout the treebank. We dropped the integer from all PP labels that in result reduced the size of the label set. A similar pattern was observed for the PP-G labels and the size of the label set was further reduced.

Another pattern was observed for VC labels. The number of common phrase labels is always one higher than the common phrase labels of the previous label. The integer part was removed from all the VC labels. The demonstrative phrase (DMP) showed a pattern that was similar to post-positional labels. The DMP labels were also simplified to reduce the labels by removing integers from them. These integers were recovered after an output was obtained from the parser. There were some patterns with NP labels, but they rather decreased the parsing scores; therefore, we utilized the four aforementioned patterns. The proposed labeling reduced the size of the tag set to 143 labels compared to 180 labels from the relative labeling.

### 4.2 DS tree representation

**Fig 6:**
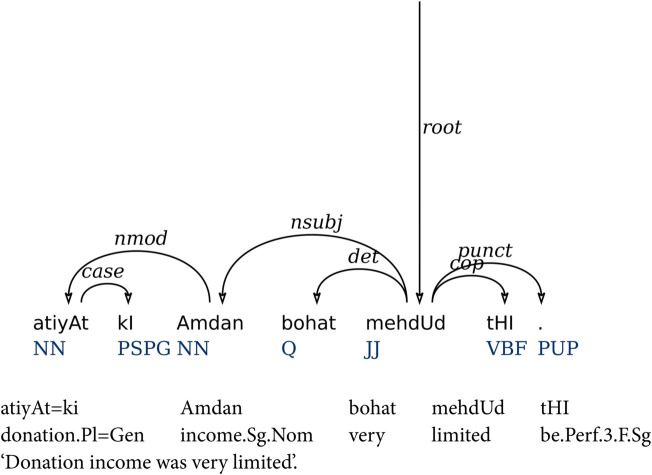
A sample DS parse tree along with its sequence labels.

DS parse trees are represented in graphical as well as CoNLL format. The graphical form uses arcs to represent dependencies which are further labeled with dependency labels as demonstrated by Fig 6. On the other hand, CoNLL format demonstrates the structure of a sentence in columns. Each token has a unique number from 1 to *n*, if there are *n* tokens in the sentence. Heads are shown by adding their token numbers and unique dependency label is assigned to each token. The CoNLL format makes it compatible to have sequential labels against tokens. A sequence labeling for the DS has been introduced in [44]. Similarly, further labeling schemes are presented in [43]. They presented four sequence labeling methods and compared the dependency parsing results. There labeling formats include: naive positional, relative positional, relative POS-based and bracketing-based labels. The relative POS-based label method outperformed the other three encodings with respect to dependency parsing.

(10) Tokens: atiyAt ki Amdan bohat
   Relative [43,44]: +1,NN,nmod -1,NN,case +1,JJ,nsubj +1,JJ,det
   mehdUd tHI .
   -1,ROOT,root
   -1,JJ,cop .
   ‘Donation income was very limited’.

Fig 6 shows a sample parse tree containing seven tokens followed by a relative POS-based encoding (10). The encoding is three tuple $\left(x_{i},p,l_{i}\right)$. *x*_*i*_ represents the relative position of the head with respect to the POS tag of the head token. It further uses the symbols  + /− to represent the direction to find a head. For example, the first token *atiyAt* ‘donations’ has the label +1,NN,nmod. Its head is *Amdan* ‘income’ that is on the right-hand side of *atiyAt* and has a POS tag NN. The label contains a  + 1 to show that the first token from right which has POS tag NN is the head of *atiyAt*. If the head is on the left side of the token *i*, then it will be represented by – symbol. We have used this encoding for both single-task and multi-task parsing experiments.

However, the naive positional encoding is two tuple $\left(x_{i},l_{i}\right)$, where *x*_*i*_ is the head token number and *l*_*i*_ is the label of token *i*. The relative positional encoding is also two tuple $\left(x_{i},l_{i}\right)$, but it generates the relative position of a head for each token. It uses symbols (+/−) to depict position of heads. Relative POS-based encoding is three tuple $\left(x_{i},p,l_{i}\right)$. It adds the relative position of a head token with respect to head’s POS tag. Finally, the bracketing-based encoding is also two tuple $\left(x_{i},l_{i}\right)$. It contains bracket symbols to represent incoming and outgoing arcs for a token *i* along with its label *l*_*i*_. We employed the relative POS-based encoding for dependency parsing that has produced state-of-the-art dependency parsing results for the CLE-UTB.

## 5 Multi-task parsing

The proposed multi-task parsing model is based on Long Short-Term Memory (LSTM) networks. We propose two main models with different features. The first model is applied as single-task learning to perform the constituency and dependency parsing independently. The second model performs multi-task learning by incorporating cross-representations and achieves improved parsing results. The following sections describe both proposed models and transfer learning paradigms.

### 5.1 Model

**Fig 7:**
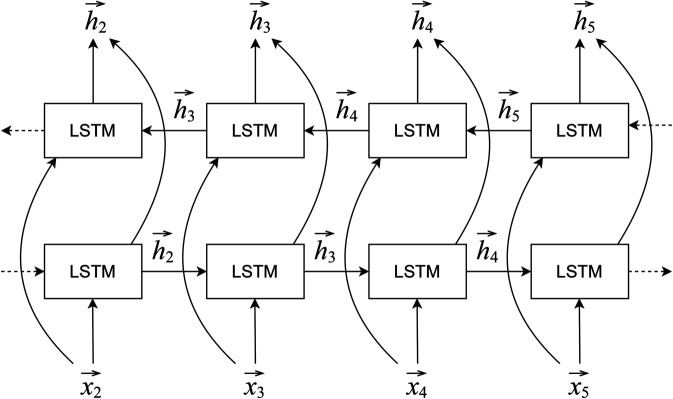
Bi-directional Long Short-Term Memory model for sequence labeling.

Recurrent neural networks (RNNs) are able to provide a learning framework to model word sequences using the entire context. Our parsing models are built on bidirectional Long Short-Term Memory (BLSTM) networks, a variant of conventional RNNs. The parse trees are converted to linear structures along with tree labels and POS tags to make them compatible with the BLSTM structure. Fig 7 shows the model architecture having two LSTM layers, one in the forward direction and the other in the backward direction.

#### 5.1.1 Single-task learning model.

**Fig 8:**
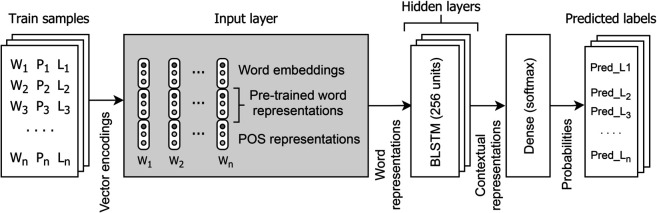
Architecture of the single-task learning model.

The single-task learning model performs constituency and dependency parsing independently. Data representation is quite similar for both types of syntactic structures; therefore, the same parsing model is trained. Fig 8 shows the architecture of the single-task learning-based parsing model.

The training set contains tokens, POS tags, and sequence labels in CoNLL format that are fed to the input layer in the form of vector encodings. Vector encodings consist of word embeddings, POS embeddings, and pre-trained word representations to perform transfer learning. At the input layer, we performed experiments with several different features to demonstrate the improvements in the parsing results. The features include character embeddings, POS tags and pre-trained word embeddings. All vector representations are concatenated to form a single vector for each token and are provided to hidden LSTM layers. Each hidden LSTM layer is set to have 256 hidden units. The contextual representations from hidden layers are then given to a dense output layer with *softmax* activation to perform the multi-class classification. The *RSMprop* optimizer is utilized with a learning rate of 0.001. The batch size is set to 32. The model was trained for 25 epochs. The parsing experiments are performed on a scientific computer cluster (https://www.scc.uni-konstanz.de).

#### 5.1.2 Multi-task learning model.

**Fig 9:**
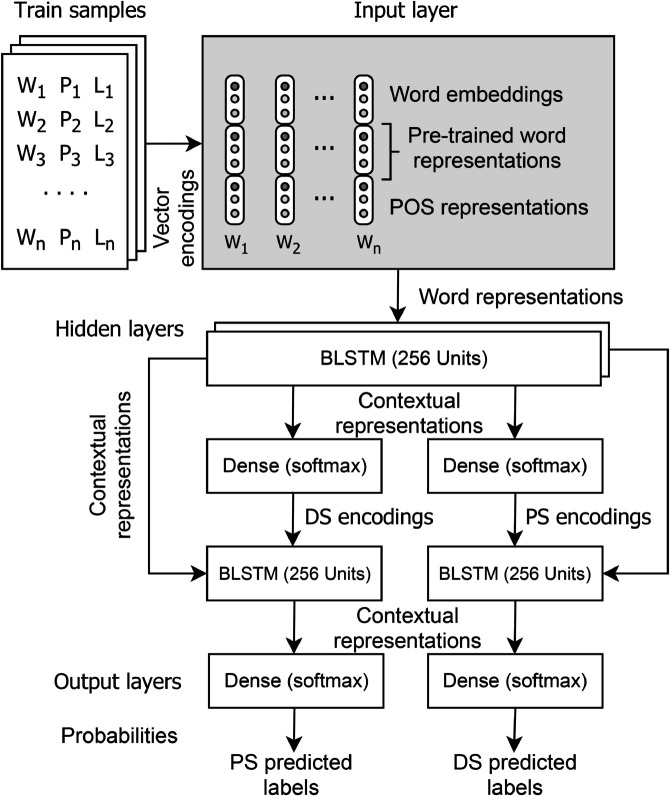
Multi-task learning based model architecture.

The multi-task learning model utilizes cross-representations to perform both types of parsing. The learned PS encodings are used for the training of the DS and vice versa. Cross-representations are quite helpful to improve the results for both constituency and dependency parsing. Fig 9 shows the architecture of the multi-task learning model.

The input layer of both single-task and multi-task learning is similar. They both use word embeddings, POS embeddings, and pre-trained word representations. Pre-trained word representations enable transfer learning that is detailed in Sect 5.2. In the multi-task learning model, we use two BLSTM hidden layers, each having 256 hidden units. The contextual representations from hidden layers are provided to two difference *softmax* layers for multi-class classification to achieve phrase and dependency encodings separately. These encodings are further fed to a single BLSTM layer along with contextual representations from the previous two BLSTM hidden layers. PS encodings are used to train DS representations, and DS encodings are used to train PS representations. Finally, two output *softmax* layers are used to predict the PS and DS labels. The *RSMprop* optimizer is used with a learning rate of 0.001 and a batch size of 32 samples is set for the training of multi-task model and the model is trained for 25 epochs.

### 5.2 Transfer learning

It is costly to create large annotated data sets for a language. Therefore, transfer learning is a suitable approach to resolve the issue of data sparsity by training word representations on large unannotated corpora. It also addresses the problem of insufficient vocabulary. To train two parsing models for Urdu, we trained word representations on a plain text corpus containing 220 million Urdu tokens collected from the Word Wide Web. The corpus has text from a number of different text genres like news, business, culture, health, sports, etc. We trained context-free word embeddings and contextualized word representations in our experiments.

#### 5.2.1 Context-free word representations.

Context-free word representations generate a single feature vector for each token in the vocabulary. In the results, a single meaning is associated with each token. However, these representations are capable of learning the syntactic and semantic details of words for a language. Well-known word representations are Word2Vec [45] and GloVe [72] embeddings among others. Both embeddings are implemented using different algorithms. Word2Vec is trained on the bases of co-occurrence of consecutive words, while GloVe embeddings are based on word-to-word co-occurrences. Word2Vec embeddings are further trained by implementing two techniques, which are the continuous bag-of-words (CBoW) and skip-gram model. The CBoW model learns the surrounding tokens of the input layer and predicts the pivotal word. On the other hand, the skip-gram is a feed-forward neural network model that learns a token from the input layer and predicts the surrounding tokens in a selected window. The size of the widow can be adjusted to enhance the contextual information. In both algorithms, a feature vector is obtained from the hidden layer. We trained Word2Vec embeddings using a skip-gram model for transfer learning in our parsing experiment. The vocabulary contained 125,000 tokens with 100 dimensions for each token. A window size of five was selected for training. Word2Vec embeddings are trained using gensim library (https://pypi.org/project/gensim). Sect 6 presents the results of the parsing models using Word2Vec word embeddings.

#### 5.2.2 Contextualized word representations.

**Fig 10:**
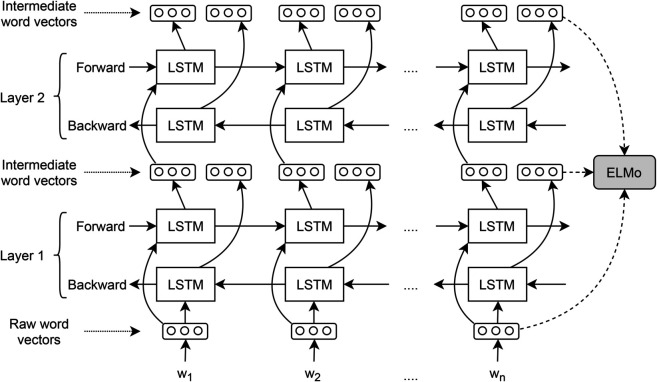
Architecture of ELMo word representation model.

In natural languages, different meanings are generally associated with words according to their context. The contextual information is quite useful for performing a sequence labeling task. Therefore, we also trained deep contextualized word representations (ELMo) (https://allennlp.org/elmo) [46] for the purpose of transfer learning. ELMo (Embeddings from Language Models) representation is helpful to achieve state-of-the-art results for many natural language processing tasks. These representations learn word vectors from deep bidirectional language models by employing character-based convolution neural networks (CNNs), which are further used to generate vectors for out-of-vocabulary (OOV) tokens as well. Character-based embeddings are also useful for learning the morphological insight of a language. The feature vectors for all tokens of a sentence are computed from pre-trained ELMo weights. These vectors use the contextual information of each token and hence a token may have different vectors according to its semantics in a sentence. Fig 10 shows the model architecture of ELMo representations.

The ELMo word vectors are computed on the top of the two-layer bidirectional language model. Each layer has two passes; the first is the forward pass and the second is the backward pass. The architecture, shown in Fig 10, uses a character-level CNN network and represents a sentence in raw word vectors at the input layer. The forward pass contains the information of a token and its context before that certain token, while the backward pass contains the information of the token and the context after that token. This contextual information forms the intermediate word vectors which are further provided to the next layer of bidirectional language model. The final representation is achieved by weighted sum of the raw vectors and two intermediate vectors for each token. However, three types of vector can be produced at each layer that are the input layer, first layer, and second layer. The final layer produces the contextual word representations that contain complete contextual information. The contextualization is useful for improving sequence labeling and to perform word sense disambiguation. The same corpus containing 220 million tokens was used to train the ELMo embeddings. We utilized word vectors produced by the last layer of the bidirectional language model in the parsing experiments.

## 6 Results and discussions

This section presents the parsing results and their interpretation. The CLE-UTB contains 7,854 sentences with 148,475 tokens. The corpus is further divided into train and test sets. The train set contains 6,881 sentences and 130,105 tokens, while the test set contains 973 sentences with 18,470 tokens. We created the test set in a way that includes the sentences from all genres of text in the corpus. Domain-wise details of the corpus are given in [16].

### 6.1 Dataset

**Fig 11:**
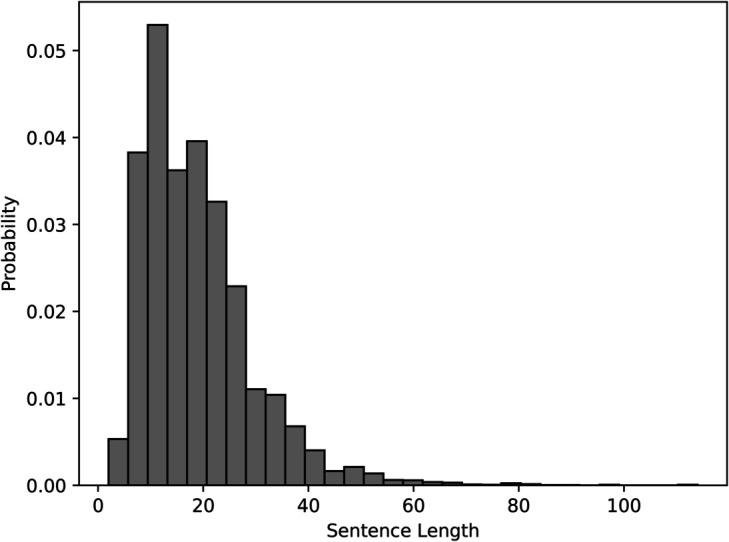
Histogram for sentence lengths in the dataset.

In this section, brief statistics of the dataset are presented. Fig 11 shows a histogram prepared with respect to sentence lengths. The x-axis shows the length groups and the y-axis presents the percentage of the sentences according to the number of tokens in them. The corpus has the highest presence of sentences with length approximately equal to ten. However, the average sentence length of the corpus is 18.9 tokens. The corpus also has sufficient coverage of longer sentences, as shown in the diagram. There are many sentences that have more than one hundred tokens in them. The histogram shows that the treebank has a natural coverage of the text with sentences of various lengths.

Table 11 shows the frequency of the phrase and functional labels in the constituency treebank. The treebank has 11 phrase labels and 10 functional labels. The frequencies in the table are arranged in decreasing order. The NP phrase has most occurrences, and the PREP phrase has lowest appearances. On the other hand, SUBJ functional label appears most of the time that marks the subject role, and the INJ label has lowest frequency that is used to annotate interjections. In general, the treebank has sufficient coverage of the core arguments of the grammar of the language.

**Table 11:** Frequencies of occurrence for phrase and function labels in the PS treebank.

Sr.#	Phrase labels	Frequency	Function labels	Frequency
1	NP	48,359	SUBJ	10,417
2	PP	21,416	G	8,923
3	S	20,791	POF	5,293
4	VC	17,530	OBJ	4,064
5	SBAR	4,741	ADJ	3,080
6	ADJP	3,331	PDL	3,040
7	ADVP	2,114	OBL	2,321
8	QP	1,136	VOC	257
9	FFP	138	VALA	213
10	DMP	57	INJ	98
11	PREP	33	—	—

Table 12 presents the frequencies of the dependency labels that appear in the generated DS treebank. In total, 27 unique dependency labels are generated in the conversion process. The case label has the highest frequency of 22,630 depicting a strong case system of the language. Urdu uses separate clitics representing cases hence the DS has additional dependency labels for each clitic. On the other hand, the fixed label has the lowest frequency. The fixed label has been used for internal case markers that have quite a few occurrences in the corpus.

**Table 12:** Frequencies of occurrence for dependency labels in the DS treebank.

Dep. label	Freq.	Dep. label	Freq.	Dep. label	Freq.	Dep. label	Freq.
case	22,630	root	7,854	acl	3,931	iobj	225
nmod	13,307	amod	7,297	advmod	3,727	discourse	203
punct	13,111	det	5,677	cop	3,011	xcomp	129
aux	11,532	conj	5,387	nummod	2,704	flat	126
obl	11,294	cc	4,516	dep	2,180	csubj	63
nsubj	10,354	mark	4,025	advcl	1,775	fixed	8
compound	9,250	obj	4,000	vocative	255	—	—

By analyzing the core arguments and labels in both treebanks, it can be concluded that the conversion is quite accurate. For example, the constituency treebank has 10,417 subjects that generate 10,354 nominal subjects (nsubj) and 63 clausal subjects (csubj) for the DS. Similarly, the constituency treebnank contains 4,064 objects that are marked with an OBJ functional label. In the generated dependency treebank, there are 4,000 direct objects marked with the obj label and there are 64 clausal objects in the constituency structure that are further mapped on the xcomp dependency label according to the third rule in Sect 3.3. However, there is a significant difference in the appearance of oblique constructions. The generated treebank contains over 11,000 occurrences of the obl label, but the constituency treebank has 2,321. The reasons for this inflation are ADJ annotations in the constituency treebank and a list of spacio-temporal nouns extracted from HUTB. According to the ninth post-conversion rule, all phrases having phrases with the ADJ functional label or having spacio-temporal nouns, are mapped on the obl label. This additional mapping significantly increased the frequency of obl in the generated treebank.

### 6.2 Evaluation metrics

To evaluate the constituency parsers, labeled recall (LR), labeled precision (LP) and labeled F-score (F1) are used. Labeled measures consider that a certain constituent is correct if tokens in constituents along with their labels are correct. Eqs 2, 3 and 4 present the formulas for the evaluation measures of the constituency parser.

$$Labeled\;Recall\;(LR)=\frac{\#\;Correct\;constituents\;in\;candidate\;set}{\#\;Constituents\;in\;reference\;set}$$ (2)

$$Labeled\;Precision\;(LP)=\frac{\#\;Correct\;constituents\;in\;candidate\;set}{\#\;Constituents\;in\;candidate\;set}$$ (3)

$$Labeled\;F-score\;(F_{1})=\frac{2.LR.LP}{LR+LP}$$ (4)

The DS contains the information of the heads and their labels at the token level; therefore, its evaluation measures are different compared to constituency parsing. The metrics used are the unlabeled attachment score (UAS), labeled attachment score (LAS), and label accuracy (LA). The unlabeled attachment score is the percentage of correct heads with respect to all tokens in the test set. The labeled attachment score is the percentage of correct heads along with correct labels, and the label accuracy is the accuracy of correct labels irrespective of the heads. The unlabeled attachment scores and label accuracy are usually higher than the labeled attachment scores. Eqs 5, 6,7 present general formulas for dependency evaluation metrics.

$$Unlabeled\;Attachment\;Score\;(UAS)=\frac{\#\;Tokens\;with\;correct\;heads}{\#\;Tokens\;in\;candidate\;set}$$ (5)

$$Labeled\;Attachment\;Score\;(LAS)=\frac{\#\;Tokens\;with\;correct\;heads\;and\;labels}{\#\;Labels\;in\;candidate\;set}$$ (6)

$$Label\;Accuracy\;(LA)=\frac{\#\;Tokens\;with\;correct\;labels}{\#\;Labels\;in\;candidate\;set}$$ (7)

### 6.3 Results

In the experiments, BLSTM-based models are trained along with various features that include character embedding, POS tags, Word2Vec embeddings, and ELMo representations. Table 13 shows the results of the constituency parsing based on single-task learning. The experiments are conducted with and without functional labels. The standalone BLSTM-based models provide baseline F-scores of 78.98 and 81.41 with and without functional labels. The F-scores demonstrate a subtle improvement when trained by utilizing character embeddings. Using POS tag encodings along with word embeddings, the results improve by five points when trained without functional labels and by about four points for functional labels.

**Table 13:** Constituency parsing results by using single-task learning paradigm.

Models & Features	No functional tags			Functional tags			
	*Rec*.	*Pre*.	$F_{1}$	*Rec*.	*Pre*.	$F_{1}$	F. acc.
BLSTM	80.94	81.89	81.41	75.47	82.83	78.98	73.72%
BLSTM+ch-emb	80.95	82.11	81.53	76.1	83.49	79.62	74.36%
BLSTM+ch-emb+pos	86.04	87.24	86.64	80.37	87.29	83.69	78.43%
BLSTM+ch-emb+pos+w2v	86.75	88.37	87.55	81.18	88.23	84.56	79.30%
BLSTM+pos+elmo	90.07	91.68	**90.86**	89.81	91.47	**90.63**	**84.37%**
BLSTM+pos+elmo+w2v	89.88	90.93	90.40	88.88	90.65	89.76	84.50%
CNN-BLSTM+pos	86.25	88.14	87.18	85.79	87.45	86.61	82.65%
CNN-BLSTM+pos+w2v	88.59	89.21	88.90	85.59	87.20	86.39	82.40%
CNN-BLSTM+pos+elmo	90.39	91.49	**90.94**	90.15	91.17	**90.66**	**84.72%**
CNN-BLSTM+pos+elmo+w2v	90.50	91.22	90.86	89.68	90.70	90.19	83.85%

We perform transfer learning by training context-free Word2Vec and contextualized ELMo word representations. In the first step, we used Word2Vec embeddings along with character encodings and POS tags. It improved the results by about 0.9 points for both cases. The ELMo representations provide character-based contextualized word embeddings, therefore, we applied ELMo representations without utilizing raw character encodings. The ELMo embeddings provide substantial improvements in the results. Without functional labels, it produces an F-score of 90.86 and an F-score of 90.63 using functional labels for the CLE-UTB. These F-scores are quite promising for a morphologically rich language. The F-scores show that ELMo embeddings are quite capable of learning the morphology and syntax of the language. We further trained the single-task model by combining ELMo and Word2Vec word embeddings, but scores started to decrease.

The second portion of Table 13, presents F-scores for a CNN-BLSTM hybrid model along with the features. The hybrid model outperforms the BLSTM models and follows a similar trend with higher F-scores. The CNN-BLSTM model that uses POS tags and ELMo embeddings provides F-scores of 90.66 and 90.94 when experimented with and without functional labels, respectively. On the other hand, the accuracy of functional labels has also increased to 84.72%. These are the state-of-the-art constituency parsing results for the CLE-UTB.

Table 14 presents the label-wise F-scores for the best performing CNN-BLSTM singe-task constituency parser. The phrase labels with higher frequencies specifically NP, PP, S and VC perform with significantly high F-scores of 89.34, 91.73, 87.82 and 98.00 respectively when evaluated without functional labels. Similarly, these phrase labels have F-scores of 89.40, 91.93, 86.01 and 98.12 with utilizing functional labels. The VC label has the highest F-scored among all others due to an intuitive verbal structure of the language. It also has high appearances in the corpus, therefore, the F-scored for VC makes a significant impact on the overall results. Table 15 shows the label-wise F scores for functional labels. The highest F-score is achieved for the ‘G’ label which is used to mark genitive post-positional phrase. Overall, frequent functional labels perform with high F-scores in single-task parsing experiments.

**Table 14:** Phrase-wise constituency parsing results by using single-task learning paradigm.

Sr.#	Phrase labels	No functional tags			Functional tags		
		*Rec*.	*Pre*.	$F_{1}$	*Rec*.	*Pre*.	$F_{1}$
1	NP	89.07	89.60	89.34	89.60	89.20	89.40
2	PP	90.66	92.82	91.73	90.81	93.08	91.93
3	S	87.19	88.47	87.82	84.40	87.67	86.01
4	VC	98.19	97.82	98.00	98.42	97.83	98.12
5	SBAR	86.11	85.96	86.03	82.33	83.48	82.90
6	ADJP	75.70	80.22	77.89	75.70	78.10	76.88
7	ADVP	89.45	91.38	90.41	89.87	88.75	89.31
8	QP	72.11	79.10	75.44	65.99	78.86	71.85
9	FFP	66.67	87.50	75.68	71.43	78.95	75.00
10	DMP	0.00	nan	nan	0.00	0.00	nan
11	PREP	0.00	0.00	nan	0.00	nan	nan

**Table 15:** Label-wise function label F-scores by using single-task learning paradigm.

Sr.#	Function labels	*Rec*.	*Pre*.	$F_{1}$
1	SUBJ	90.01	86.81	88.38
2	G	99.44	99.16	99.30
3	POF	83.64	83.26	83.45
4	OBJ	63.17	66.75	64.91
5	ADJ	84.22	85.37	84.79
6	PDL	78.61	85.0	81.68
7	OBL	41.31	47.56	44.21
8	VOC	68.97	86.96	76.92
9	VALA	95.24	86.96	90.91
10	INJ	100.0	100.0	100.0

**Fig 12:**
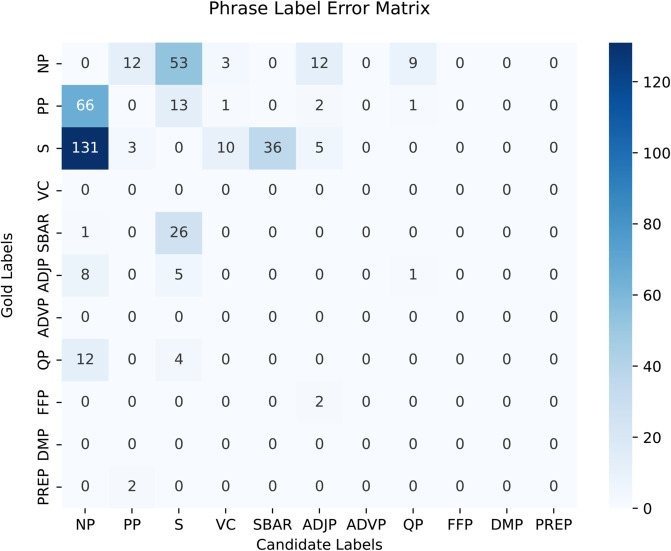
Phrase label error matrix based on single-task learning paradigm.

Fig 12 shows the error matrices for the best performing single-task learning parser for the phrase labels. It is important to note that the diagram shows only overlapping predicting labels in the gold and candidate labels. The x-axis has labels from candidate set and the gold labels on the y-axis that are present in the evaluation set. The highest confusions are with the ‘S’ and NP followed by the PP and NP labels. PP and NP are closely related in the annotations as each PP constituent contains an NP in it. In the same way, there are confusions between the labels NP and ‘S’ due to their syntactic similarities.

**Fig 13:**
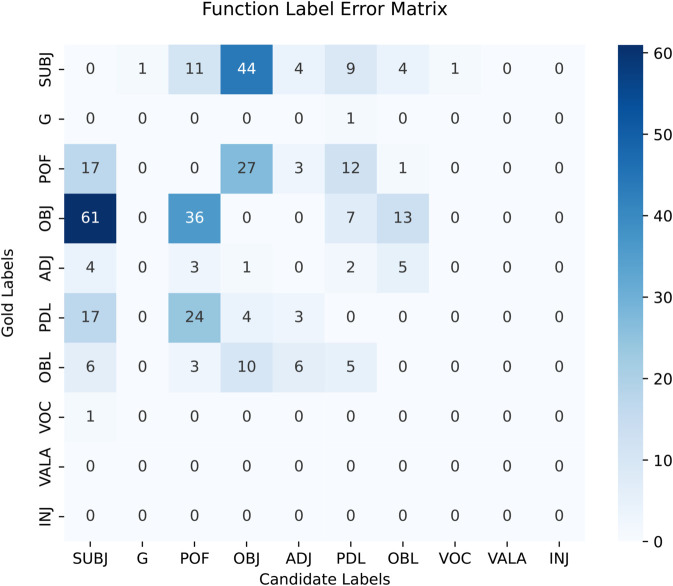
Function label error matrix based on single-task learning paradigm.

Furthermore, Fig 13 presents confusions among functional labels when predicted by training single-task CNN-BLSTM hybrid model. The highest confusions are between subjects and objects due to the syntactic resemblances. The second largest differences are between objects and POF labels. The Urdu language has a flexible word-order having SOV as the most frequent order with pre-verbal position of objects [15]. Similarly, the POF mostly has pre-verbal position that is used to annotate complex predicate structures. This confusion is due to the positional similarity of OBJ and POF. Another prominent disagreement is between POF and PDL. The PDL label is used to annotate copula constructions that usually have pre-verbal positions producing these confusions [16]. However, there are some other minor confusions in the set of predicted candidates. Overall, the constituency parsing results and functional label accuracy are quite promising for the CLE-UTB.

**Fig 14:**
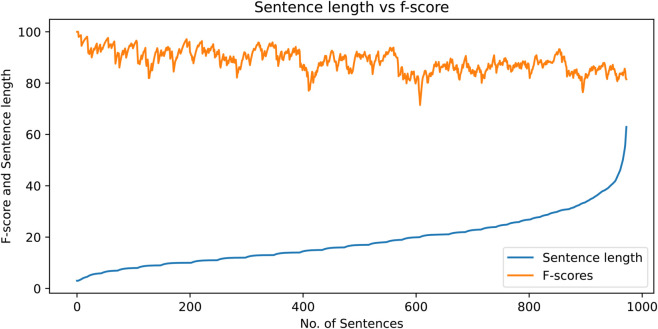
Constituency parsing results with respect to sentence length.

Fig 14 presents a graph showing constituency parsing results with respect to sentence lengths. Constituency parsers perform better on shorter sentences, indicating accurate predictions when the syntactic complexity is relatively low, which is consistent with previous studies [15]. Sentence lengths are represented with the blue line, and F-scores are represented by an orange color. There is a gradual decline in the F score as the length of the sentence increases. Despite increased structural complexity and embedding depth, this degradation remains moderate. It is important to note that our parser maintains high performance even for longer sentences, suggesting a strong generalization capability of parsing models and the proposed labeling scheme. This behavior also demonstrates that contextualized representations and training techniques effectively handle the linguistic richness of Urdu, even in structurally dense sentences.

Table 16 presents the results of the dependency parsing based on single task learning. As the single-task learning model is similar for both types of syntactic structures, the same features and parameters are used in the training. The labeled attachment score (LAS) is important as it computes the correctness of heads as well as labels. The baseline BLSTM model provides an LAS of 74.28 that is further improved by character embeddings by a point of 0.2. However, POS tags provide a significant improvement of more than eight points with an LAS of 82.55. Word2Vec embeddings further increase LAS scores to 83.59. Using the ELMo embedding, LAS has been enhanced to 85.13 which is quite promising for the converted dependency treebank. Unlike the constituency parser, the dependency parser shows an increase of LAS when trained by combining both word embedding and produces a state-of-the-art LAS of 85.36 on the CLE-UTB. The results outperform the LAS of 81.6 from the famous MatlParser [17,50] and the LAS of 84.2 from the transition-based BLSTM BIST-Parser [17,39]. The CNN-BLSTM hybrid model also produces competitive results; however, for dependency parsing, the BLSTM model performs better.

**Table 16:** Dependency parsing results by using single-task learning paradigm.

Models & Features	*UAS*	*LAS*	*LA*
BLSTM	79.51	74.28	87.33
BLSTM + ch-emb	79.64	74.48	87.16
BLSTM + ch-emb + pos	86.75	82.55	91.45
BLSTM + ch-emb + pos + w2v	87.59	83.59	92.05
BLSTM + pos + elmo	88.75	85.13	92.84
BLSTM + pos + elmo + w2v	88.94	**85.36**	93.11
CNN-BLSTM + pos	86.83	82.49	91.32
CNN-BLSTM + pos + w2v	87.41	83.42	92.01
CNN-BLSTM + pos + elmo	88.74	85.05	92.85
CNN-BLSTM + pos + elmo + w2v	88.87	**85.09**	92.84

Table 17 shows the label-wise F-scores for the best performing single-task dependency parser. The scores for the frequent labels are quite promising. However, labels with rare occurrences in the evaluation set such as csubj and fixed cannot contribute to overall LAS. The csubj has ten samples and the fixed does not appear in the evaluation set. It is important to note that supervised methods, specifically techniques based on Deep Neural Networks, are able to perform many NLP tasks but require a substantial amount of training and evaluation data. In our work, both constituency and dependency parsers perform less effective predictions on rarely occurring constructions due to data sparsity.

**Table 17:** Label-wise labeled attachment scores (LAS) for dependency parsing using single-task learning paradigm.

Sr.#	Dependency labels	*Rec*.	*Pre*.	$F_{1}$
1	acl	75.26	69.96	72.51
2	advcl	64.35	78.09	70.56
3	advmod	82.05	80.58	81.31
4	amod	88.48	88.02	88.25
5	aux	96.39	96.73	96.56
6	case	98.66	98.79	98.72
7	cc	83.42	83.13	83.27
8	compound	84.88	83.68	84.28
9	conj	68.40	69.5	68.95
10	cop	81.51	81.3	81.40
11	csubj	0.00	0.00	nan
12	dep	88.01	91.09	89.52
13	det	92.71	90.26	91.47
14	discourse	76.92	71.43	74.07
15	fixed	0.00	0.00	nan
16	flat	70.00	70.00	70.00
17	iobj	36.84	26.92	31.11
18	mark	91.38	90.86	91.12
19	nmod	77.46	80.34	78.87
20	nsubj	77.43	81.13	79.24
21	nummod	79.87	81.70	80.77
22	obj	70.28	60.45	65.00
23	obl	83.51	83.57	83.54
24	punct	78.27	78.17	78.22
25	root	89.21	87.06	88.12
26	vocative	62.5	66.67	64.52
27	xcomp	41.18	50.00	45.16

Table 18 further presents the error analysis for dependency labels for the best performing single-task learning model. Due to a large number of labels, the labels with 10 or higher confusions are shown in the table. The highest confusions are among nominal subjects and direct objects because of their syntactic resemblance. The second and third highest confusions are among the labels nmod, nsubj and obj. The nmod label presents nominal modifiers and NPs within post-positional phrases representing genitive cases are mapped on the nmod label (Table 4). The mapping creates syntactic similarity with nominal subjects and direct objects in the treebank. Similarly, the nmod also has significant confusions with the compound label. Complex predicate structures are mapped on the compound label and the higher portion of the complex predicate structures consists of NPs, hence resulting in these confusions by the parser. There are some minor confusions among the other dependency labels.

**Table 18:** Confusion matrix of top 10 or more errors for dependency parsing using single-task learning paradigm.

Gold	Cand	Freq.	Gold	Cand	Freq.	Gold	Cand	Freq.
nsubj	obj	82	nmod	obl	31	obj	obl	15
nmod	nsubj	50	cop	root	29	obl	root	15
nmod	obj	49	obj	nmod	29	nmod	conj	14
nmod	compound	40	obl	obj	23	root	compound	14
advcl	acl	37	obl	nmod	22	acl	conj	13
compound	nmod	37	compound	nsubj	19	advcl	conj	13
compound	obj	36	amod	compound	17	cop	acl	12
root	cop	36	compound	amod	17	root	nsubj	12
obj	nsubj	35	nummod	det	17	nsubj	obl	11
nsubj	compound	33	conj	root	16	obl	compound	11
nsubj	nmod	33	conj	acl	15	compound	root	10
obj	compound	33	nsubj	root	15	iobj	obj	10

**Fig 15:**
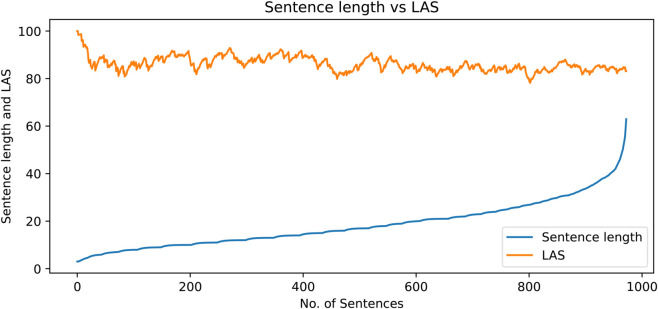
Dependency parsing results with respect to sentence length.

Unlike the constituency parser, the dependency parser performs quite uniformly for sentences of various lengths, as shown in Fig 15. The orange line is straighter compared to Fig 14 depicting the ability of the parser to perform similar for shorter and longer sentences.

The single-task learning-based parsers produce state-of-the-art results for both constituency and dependency structures for the CLE-UTB. However, a multi-task learning-based model is developed to enhance the parsing results by learning cross-structure representations. Table 19 presents the results of the multi-task learning paradigm trained without utilizing functional labels. Both constituency and dependency parsing results show an upward trend even for the baseline model. The baseline model produces an F-score of 82.49 as compared to 81.41 and an LAS of 74.57 as compared to 74.28 from single-task learning paradigm. Similarly, the feature set further enhances the parsing scores. The multi-task model enhances the F-score for the CLE-UTB to 90.99 when trained with the ELMo Embeddings by training the BLSTM parser. The CNN-BLSTM hybrid model further improves the performance and produces a best F-score of 91.39 for constituency parsing. Without using functional labels, the highest dependency LAS is 85.41 when trained with context-free and contextualized word representations together. It is quite clear that the constituency parsing scores are improved from 90.94 to 91.39 by learning the dependency encodings trained and evaluated without functional labels. There is also a subtle improvement in the dependency results with an LAS of 85.41. However, functional labels could be more useful as they encode the grammatical roles on PS parse trees.

**Table 19:** Multi-task learning based constituency and dependency parsing results without using functional tags.

Models & Features	*Rec*.	*Pre*.	$F_{1}$	*UAS*	*LAS*	*LA*
BLSTM	81.18	83.84	82.49	79.91	74.57	87.73
BLSTM+ch-emb	81.53	83.46	82.48	79.81	74.87	88.17
BLSTM+ch-emb+pos	86.49	88.43	87.45	87.01	82.79	91.5
BLSTM+ch-emb+pos+w2v	87.03	89.08	88.04	87.52	83.42	91.81
BLSTM+pos+elmo	90.38	91.62	90.99	88.3	84.45	92.51
BLSTM+pos+elmo+w2v	89.22	91.37	90.28	88.69	85.09	92.87
CNN-BLSTM+pos	88.59	89.23	88.91	87.26	82.89	91.45
CNN-BLSTM+pos+w2v	88.31	89.80	89.05	87.54	83.36	91.76
CNN-BLSTM+pos+elmo	90.67	92.12	**91.39**	88.75	84.93	92.79
CNN-BLSTM+pos+elmo+w2v	90.17	92.13	91.14	89.13	**85.41**	92.92

Table 20 presents the multi-task learning-based parsing scores by utilizing functional labels in experiments. The CNN-BLSTM hybrid model produces a best LAS of 85.47 when trained with Word2Vec and ELMo embeddings. The constituency results are not much improved when trained with functional labels as compared to single-task learning model. The functional labels encode the dependency information in the PS trees therefore, the presence of functional labels in the constituency structure parse trees does not improve leaning capability of the model. On the other hand, functional labels contribute in the generation of larger set of sequential labels. However, the accuracy of the functional labels improved significantly incorporating DS-based encodings in the model. The accuracy for functional labels is enhanced by 1.51 points from 84.72% to 86.23%. Overall, the constituency parsing is improved by using DS encodings trained without using functional labels and the dependency parsing results are enhanced by incorporating constituency structure encodings along with functional labels.

**Table 20:** Multi-task learning based constituency and dependency parsing results by using functional tags.

Models & Features	*Rec*.	*Pre*.	$F_{1}$	F. acc.	*UAS*	*LAS*	*LA*
BLSTM	77.68	82.3	79.92	75.15%	79.80	74.70	87.84
BLSTM+ch-emb	78.97	84.17	81.49	76.72%	79.77	74.76	88.12
BLSTM+ch-emb+pos	82.73	87.89	85.23	80.46%	87.14	82.93	91.62
BLSTM+ch-emb+pos+w2v	83.41	88.55	85.90	81.13%	87.42	83.38	91.93
BLSTM+pos+elmo	86.97	91.67	89.26	84.49%	88.75	85.23	92.98
BLSTM+pos+elmo+w2v	85.47	91.18	88.23	83.46%	89.08	85.47	92.98
CNN-BLSTM+pos	86.63	88.63	87.61	83.43%	87.06	82.88	91.58
CNN-BLSTM+pos+w2v	87.85	89.67	88.75	83.78%	87.76	83.60	91.95
CNN-BLSTM+pos+elmo	90.37	91.78	91.07	86.00%	88.68	84.90	92.73
CNN-BLSTM+pos+elmo+w2v	90.32	91.86	**91.08**	**86.23%**	89.28	**85.69**	93.20

Tables 21 and 22 show label-wise constituency and dependency parsing results achieved from the best performing multi-task learning models. The parsing scores for the core and frequent labels have been enhanced by utilizing cross-structure encodings. For the constituency structure, improvements are observed for NP, PP, ADJP and DMP labels with increase of 0.75, 0.84, 2.32 and 40.0 points respectively. Similarly, for the DS, prominent improvements are analyzed for some of the core labels nsubj, obj, nmod and iobj with increase of 1.53, 0.71, 0.64 and 7.60 points respectively.

**Table 21:** Label-wise constituency parsing results from the multi-task learning paradigm.

Sr.#	Phrase labels	No functional tags			Functional tags		
		*Rec*.	*Pre*.	$F_{1}$	*Rec*.	*Pre*.	$F_{1}$
1	NP	89.98	90.2	90.09	89.52	90.02	89.77
2	PP	91.14	94.04	92.57	90.56	93.23	91.87
3	S	86.13	89.10	87.59	85.58	88.10	86.82
4	VC	98.7	98.47	98.58	98.47	97.92	98.19
5	SBAR	83.36	86.02	84.67	83.02	84.76	83.88
6	ADJP	78.26	82.26	80.21	77.49	82.79	80.05
7	ADVP	91.14	89.26	90.19	91.14	88.16	89.63
8	QP	70.75	76.47	73.5	62.59	83.64	71.60
9	FFP	61.9	92.86	74.29	71.43	100	83.33
10	DMP	20.0	20.0	20.0	40.0	40.0	40.0
11	PREP	0.00	nan	nan	0.00	nan	nan

**Table 22:** Label-wise LAS for dependency parsing from the multi-task learning paradigm.

Dep. labels	*Rec*.	*Pre*.	$F_{1}$	Dep. labels	*Rec*.	*Pre*.	$F_{1}$
acl	74.23	70.35	72.24	fixed	0.00	nan	nan
advcl	64.81	78.21	70.88	flat	70.00	58.33	63.63
advmod	81.59	82.91	82.24	iobj	31.58	50.00	38.71
amod	87.85	89.64	88.74	mark	90.23	89.54	89.88
aux	96.53	96.6	96.56	nmod	77.99	81.09	79.51
case	98.28	98.15	98.21	nsubj	82.61	79.01	80.77
cc	83.60	83.9	83.75	nummod	82.11	87.12	84.54
compound	86.65	82.61	84.58	obj	64.26	67.23	65.71
conj	72.99	71.63	72.30	obl	83.51	84.29	83.90
cop	80.73	81.58	81.15	punct	79.81	79.55	79.68
csubj	0.00	nan	nan	root	89.31	87.69	88.49
dep	88.39	91.83	90.08	vocative	68.75	75.86	72.13
det	94.29	91.03	92.63	xcomp	52.94	52.94	52.94
discourse	69.23	47.37	56.25	—	—	—	—

**Fig 16:**
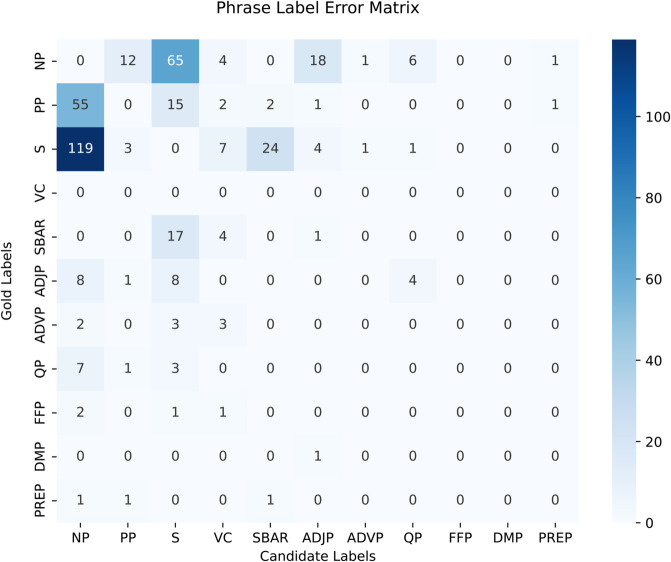
Phrase label error matrix based on the multi-task learning paradigm.

**Fig 17:**
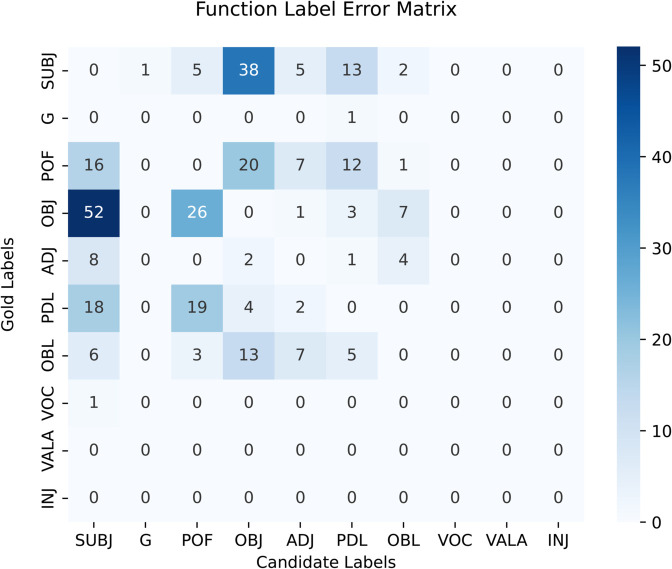
Function label error matrix based on the multi-task learning paradigm.

Figs 16 and 17 demonstrate the enhancement of the parsing results by showing reduced confusions between phrase and functional labels. The errors between ‘S’ and NP labels are reduced from 131 to 119. Similarly, the confusions between PP and NP labels are decreased from 66 to 55. However, an increase of errors is observed between NP and ‘S’ labels, overall, the multi-task learning paradigm is helpful to achieve improved results with minimized confusions between labels. Similarly, the difference of functional labels in the evaluation set are reduced. The highest disagreement was observed between OBJ and SUBJ with a value of 61 that is reduced to 52 and the confusions between SUBJ and OBJ are minimized from 44 to 38. The label-wise errors are reduced for all the functional labels by employing multi-task learning models with a reduction of 12.4%.

Table 23 shows confusions for the dependency parser for ten or higher disagreements. The highest confusions of 82 were observed with the nsubj and obj label that are reduced to 37 in the multi-task learning paradigm. Some of the confusion values show the differences in the opposite direction. However, total number of disagreements in the top 36 labels are reduced from 901 to 861 with an error reductions of 4.4%.

**Table 23:** Confusion matrix of top 10 or more errors for dependency parsing by Multi-task learning paradigm.

Gold	Cand	Freq.	Gold	Cand	Freq.	Gold	Cand	Freq.
obj	nsubj	59	nsubj	nmod	27	compound	root	14
nmod	nsubj	57	cop	root	26	conj	root	14
nmod	compound	44	compound	obj	25	cop	acl	14
nsubj	obj	37	obl	nmod	25	advcl	conj	14
obj	compound	37	compound	nsubj	24	obl	root	13
advcl	acl	37	amod	compound	23	obl	nsubj	13
nmod	obl	37	obl	obj	22	acl	conj	12
root	cop	35	nummod	det	18	obj	obl	11
obj	nmod	35	root	nsubj	17	nsubj	obl	10
nmod	obj	32	root	compound	16	acl	advcl	10
compound	nmod	28	nsubj	root	15	nmod	conj	10
nsubj	compound	27	obl	compound	14	acl	cop	9

We conducted multiple parsing experiments by employing single-task and multi-task learning paradigms. Single-task learning models are trained for the constituency and dependency parsing independently. Various features are incorporated with the single-task learning models and state-of-the-art parsing results are achieved for the CLE-UTB. Furthermore, multi-task learning models are trained to incorporate cross-structure representations in the models. The cross-structure encodings were quite helpful to enhance parsing results. The experiments are conducted by including the functional labels as well as without them. The constituency parsing results are enhanced by utilizing the dependency encodings in the models. However, the dependency parsing scores are enhanced by incorporating the functional labels in the constituency treebank. The accuracy of the functional labels is also improved by the multi-task learning models. Overall, the parsing results are evident that learning cross-structure representations are quite helpful for parsing task. The contextualized word representations are also helpful in producing improved results by performing transfer learning for small to medium-sized annotated data sets. We conducted multiple parsing experiments by employing single-task and multi-task learning paradigms. Single-task learning models are trained for the constituency and dependency parsing independently. Various features are incorporated with the single-task learning models and state-of-the-art parsing results are achieved for the CLE-UTB. Furthermore, multi-task learning models are trained to incorporate cross-structure representations in the models. The cross-structure encodings were quite helpful to enhance parsing results. The experiments are conducted by including the functional labels as well as without them. The constituency parsing results are enhanced by utilizing the dependency encodings in the models. However, the dependency parsing scores are enhanced by incorporating the functional labels in the constituency treebank. The accuracy of the functional labels is also improved by the multi-task learning models. Overall, the parsing results are evident that learning cross-structure representations are quite helpful for parsing task. The contextualized word representations are also helpful in producing improved results by performing transfer learning for small to medium-sized annotated data sets.

## 7 Conclusion

This work presents two syntactic parsing paradigms to parse a morphologically rich language Urdu. First, the single-task learning paradigm along with several features including character embeddings, POS tags, context-free and contextualized word representations. Second, the multi-task learning paradigm that performs the parsing task by learning cross-structure representations. A PS treebank (CLE-UTB) is transformed to the DS. A language-dependent head-word model and a constituency to dependency label mapping scheme are devised followed by several post-conversion rules. The single-task learning paradigm produced state-of-the-art constituency and dependency parsing results for the CLE-UTB when trained with contextualized word representations. The character-based contextualized representations are quite capable of learning the morphology and syntactic information of the language. The multi-task learning paradigm further enhanced the parsing results by learning cross-structure representations. The results are evident that cross-structure encodings are useful to improve the syntactic parsing.
