# Correction: Multi-task learning by using contextualized word representations for syntactic parsing of a morphologically rich language

**DOI:** 10.1371/journal.pone.0339262

**Published:** 2025-12-17

**Authors:** Toqeer Ehsan, Miriam Butt, Sarmad Hussain, Hassan Alhuzali, Ali Al-Laith

In the Funding statement, the grant number from the funder Umm Al-Qura University, Saudi Arabia is listed incorrectly. The correct grant number is: 25UQU4320430GSSR03.
